# Non-invasive detection of VEGF secretion from small clusters of mesenchymal stem cells using VEGF-SSSA integrated into the CellStudio platform

**DOI:** 10.1007/s00604-025-07319-2

**Published:** 2025-07-05

**Authors:** Enrique Azuaje-Hualde, Naiara Lartitegui-Meneses, Fernando Benito-Lopez, Lourdes Basabe-Desmonts

**Affiliations:** 1 Microfluidics Cluster UPV/EHU, BIOMICs Microfluidics Group, University of the Basque Country UPV/EHU, Vitoria-Gasteiz, Spain; 2 Microfluidics Cluster UPV/EHU, Analytical Microsystems & Materials for Lab-On-a-Chip (AMMa-LOAC) Group, University of the Basque Country UPV/EHU, Vitoria-Gasteiz, Spain; 3 Basque Foundation of Science, IKERBASQUE, Bilbao, Spain

**Keywords:** Aptasensor, Live-cell analysis, Cell secretion, Microenvironment, Analytical platforms, Cell behavior

## Abstract

Accurately detecting cell secretions in complex microenvironments is crucial for understanding cellular communication, disease progression, and therapeutic responses. Traditional methods, such as ELISA, provide limited insight into the spatiotemporal dynamics of secretions, often requiring invasive or endpoint analyses. To address these challenges, we have functionalized the CellStudio platform—previously developed to integrate cell adhesion areas and microbeads patterns—with a novel self-reporting structure-switching signaling aptamer (SSSA) for vascular endothelial growth factor (VEGF) detection. This functionalization enables in situ single-step, real-time quantification of VEGF secretion from hundreds of live mesenchymal stem cell (MSC) clusters over a 24-h period, using standard fluorescence microscopy without the need for cell fixation. The spatial resolution provided by this system enables precise, localized monitoring of secretion events without the need to add extra reagents. This user-friendly platform is easily adaptable to conventional laboratory workflows, offering a versatile tool with potential for studying cellular behavior in real time with minimal technical barriers.

**Graphical abstract:**

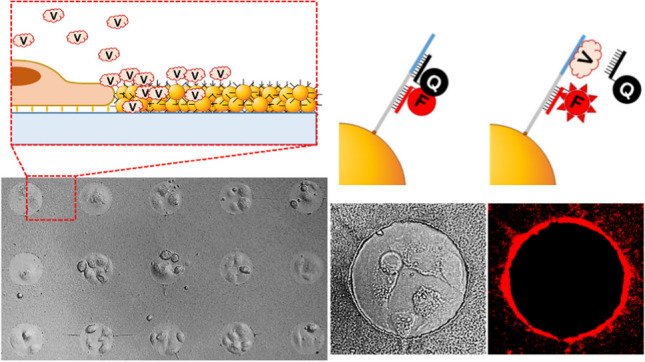

## Introduction

The study of cell secretion is fundamental for understanding intercellular communication and physiological processes such as immune responses and tissue regeneration [1, 2]. However, traditional detection methods, such as enzyme-linked immunosorbent assays (ELISA), rely on bulk supernatant collection, which provides only averaged data across large cell populations and requires additional equipment beyond the cell culture plate. These approaches lack spatial and temporal resolution. Recent advancements have introduced alternative techniques capable of in situ and even real-time analysis of cellular secretion, including droplet microfluidic and nanoplasmonic platforms that position biosensors in close proximity to cells [3–7]. These systems, such as those involving antibody-functionalized microchambers or nanohole plasmonic substrates, offer improved resolution and sensitivity but often involve complex fabrication and operation steps. As a result, their implementation can be limited by technical demands and reduced flexibility in adapting to diverse biological setups.

Aptamers, particularly structure-switching signaling aptamers (SSSAs), have emerged as powerful tools for biosensing applications due to their high stability, small size, and customizable sequences [8–10]. SSSAs function by undergoing strand displacement upon target recognition, triggering a detectable signal, typically fluorescence [11–13]. These aptamer-based biosensors have been successfully used to detect pharmaceuticals, growth factors, and cytokines [14–18]. Their ability to generate a direct fluorescence response without requiring additional labeling or surface modifications makes them ideal for integration into live-cell analysis platforms. Unlike traditional immunoassays, which require multiple processing steps and often involve endpoint detection, SSSAs provide a single-step approach that can enable continuous monitoring of secretion events [19].

Despite their advantages, very few studies have successfully integrated SSSAs into analytical platforms in direct proximity to cells. Notable examples include the work of Zhou et al. [20], who utilized SSSAs to detect Transforming growth factor-β (TGF-β) secreted from hepatocytes within a microfluidic device, and Zhao et al. [21], who developed SSSA-modified cells to probe platelet-derived growth factor (PDGF) on the surface of mesenchymal stem cells (MSCs). These studies highlight the potential of SSSA-based biosensors but also reveal the scarcity of integrated platforms that fully exploit their capabilities. To maximize their enhanced sensing performance, it is essential to integrate SSSAs into platforms specifically designed for monitoring cell secretion.

**Fig. 1:**
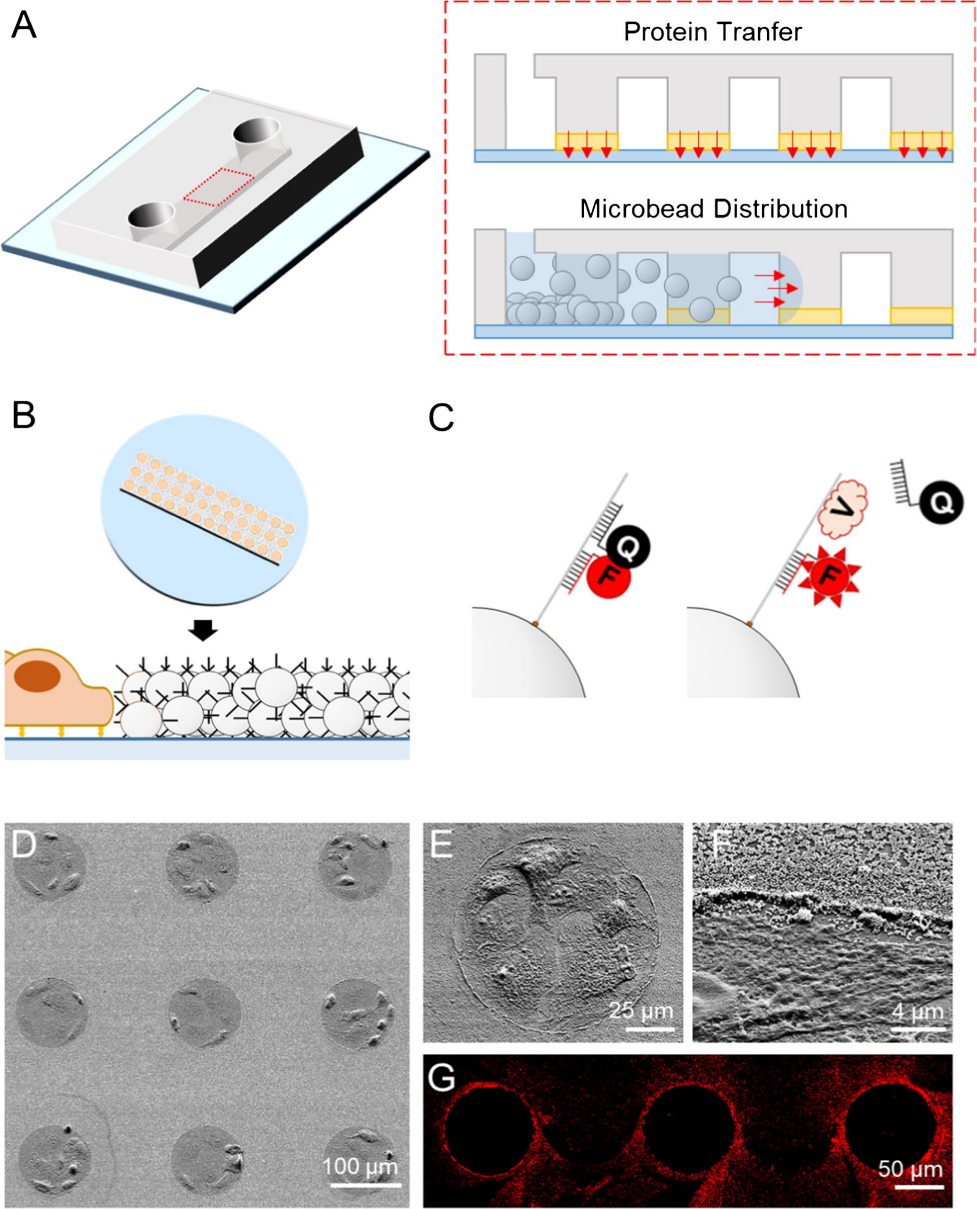
SSSA-based detection using CellStudio substrates. **A** Schematic illustration of the printing and vacuum lithography process used for fabricating CellStudio substrates. A single PDMS slab is utilized to facilitate protein transfer via microcontact printing, allowing the replication of 2D protein patterns in the contact area between individual pillars and the substrate surface. Vacuum-driven flow then enables the uniform distribution of microbeads suspended in the fluid between the pillars. **B** Illustration of a CellStudio substrate featuring an array of cell clusters, each surrounded by microbeads in their immediate vicinity, secreting VEGF (V). **C** VEGF-SSSA-functionalized microbeads: The interaction of VEGF (V) with the aptamer sequence induces a conformational change in the double-stranded DNA, separating the quencher from the fluorophore and generating a fluorescence signal only when VEGF binds to the SSSA. **D**–**F** Scanning electron microscope (SEM) images at different magnifications of CellStudio substrates, showing MSC clusters. **G** Fluorescence microscopy image showing three adjacent dots, illustrating the interaction between VEGF-SSSA and secreted VEGF

To address the need for integrated systems capable of advanced biosensing, we previously developed CellStudio [22], a versatile and modular biosensing platform that combines printing and vacuum lithography (PnVlitho) [23] with bead-based biosensors [24, 25]. Designed to facilitate the detection of cell-secreted factors, CellStudio precisely positions biosensors near small cell clusters, allowing spatially resolved secretion analysis. CellStudio substrates are fabricated through a two-step process using a single polydimethylsiloxane (PDMS) slab, combining microcontact printing with degas-driven particle flow between PDMS features (Fig. 1A). This method enables the formation of a two-dimensional pattern of protein dots surrounded by a three-dimensional microbead arrangement. The platform supports hundreds of independent cell clusters on a single substrate, with each cluster anchored to a protein adhesion dot that ensures controlled cell attachment and uniform patterning [26–28]. The surrounding three-dimensional microbead network can be functionalized with bioreceptors (Fig. 1B), enabling high-resolution detection of cell-secreted molecules using standard fluorescence microscopy. This adaptable system allows researchers to regulate factors such as cell adhesion, spatial distribution, and cell–cell contact, making it a versatile tool for studying various biological models and experimental conditions.

Despite these advantages, previous validations of the CellStudio platform relied on commercial sandwich immunoassays, which require endpoint fixation for labeling secreted factors. This limitation prevents live-cell monitoring and makes it difficult to capture secretion dynamics over time.

In this study, an innovative approach that integrates CellStudio with structure-switching signaling aptamers (SSSAs) to enable high-resolution, real-time monitoring of cell secretion, overcoming the limitations of traditional biosensing methods, is presented. Specifically, we functionalized microbeads within the CellStudio platform with a SSSA previously developed by our group [29], enabling the detection of cell-secreted vascular endothelial growth factor (VEGF) in real time. VEGF is a key growth factor involved in angiogenesis, vasculogenesis, wound healing, and tissue remodeling [30–32]. This integration enables single-step, high-sensitivity detection of VEGF secretion from MSC clusters, eliminating the need for fixation or supernatant collection (Fig. 1C). The system allows for simultaneous analysis of hundreds of MSC clusters, each consisting of just 4 to 5 cells, on a single platform (Fig. 1D–G). By incorporating VEGF-specific SSSA aptasensors (VEGF-SSSA) into the bead-based assay, we enable direct, single-step detection of VEGF secretion from live MSC clusters, eliminating the need for fixation or supernatant collection, while simultaneously allowing visualization of cell morphology through standard fluorescence microscopy.

## Experimental section

### VEGF-SSSA solutions

Biotinylated VEGF-SSSA constructs were designed in-house based on previous reports [29]. VEGF-SSSA sequences were purchased from Integrated DNA Technologies (IDT, Belgium). Three oligonucleotide sequences were obtained: 5′- TGTGGGGGTGGACTGGGTGGGTACCGTCACTCGCCTCGCACCGTCC–Biotin-3′ (aptamer sequence), 5′-GGACGGTGCGAGGCG-Cy5Sp-3′ (fluorescence sequence), and 5′–IabRQ-GTGACGGTACCC-3′ (quencher sequence). The fluorophore (Cy5) has an excitation wavelength of 648 nm and an emission wavelength of 668 nm. The quencher (Iowa Black RQ) has an absorbance between 500 and 700 nm.

**Fig. 2:**
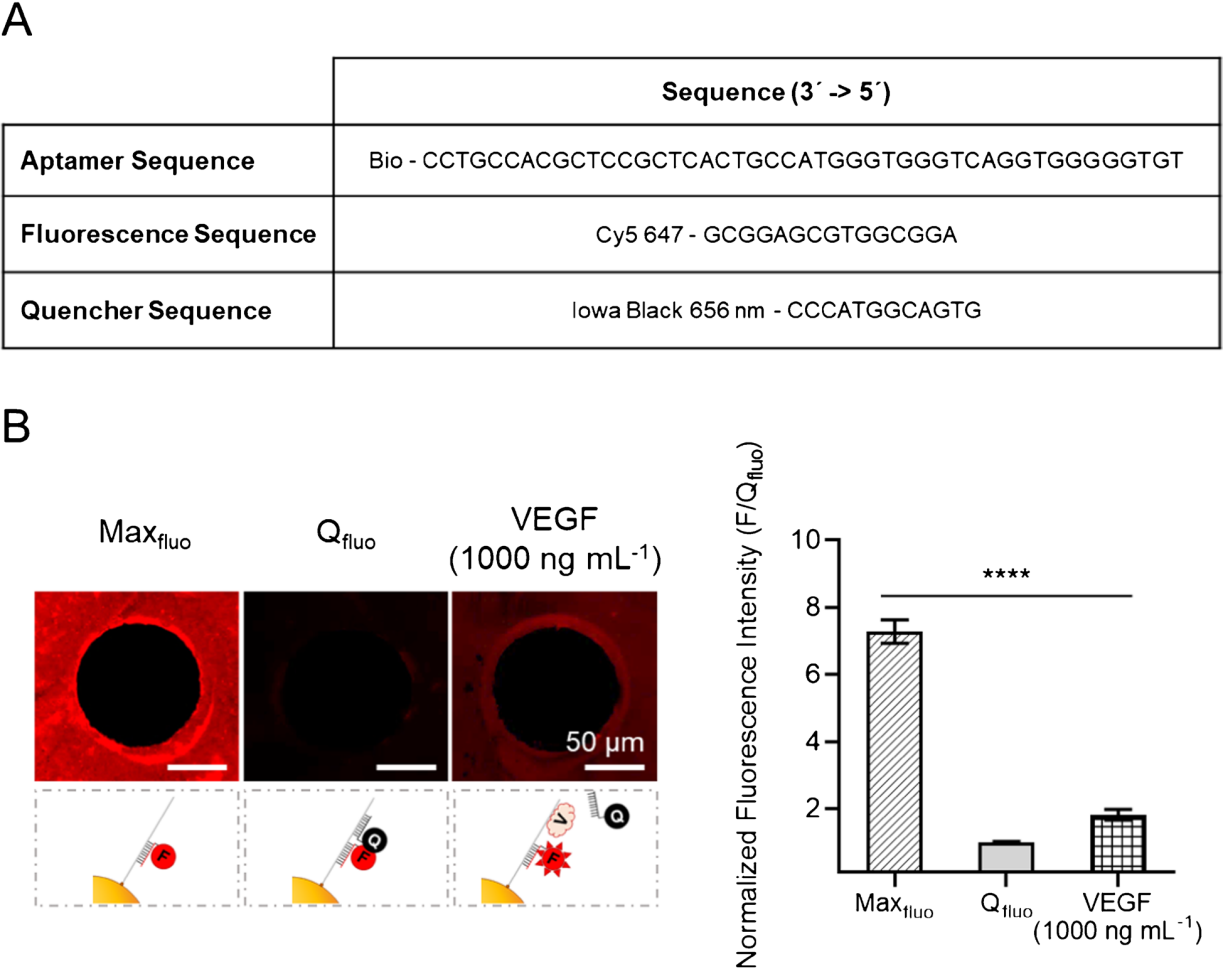
Characteristics of the VEGF-SSSA. **A** Table listing the sequences comprising the VEGF-SSSA. **B** Fluorescence microscopy images (left) and corresponding fluorescence intensity plot (right) illustrating the different fluorescence states of the microbeads pattern functionalized with the VEGF-SSSA construct. Shown are the maximum fluorescence in the absence of the quencher sequence (Max_Fluo_), the quenched minimum fluorescence when the full construct is formed (Q_Fluo_), and the fluorescence recovery of the full VEGF-SSSA upon incubation with 1000 ng mL⁻^1^ VEGF. All data was normalized with the mean value of the quenched VEGF-SSSA (Q_Fluo_). Error bars mean SEM (*n* = 100 dots from 3 samples). Statistical significance: non-parametric Kruskal-Wallis test (****p ≤ 0.001)

The VEGF-SSSA solution was prepared by mixing 2 nM aptamer sequence, 6 nM fluorescence sequence, and 6 nM quencher sequence in a saline buffer containing 100 mM sodium chloride, 5 mM potassium chloride, and 2 mM TRIS in distilled water, adjusted to pH 7. For system characterization, an additional solution without the quencher sequence was prepared (VEGF-SSSA-Max_fluo_), consisting of 2 nM aptamer sequence and 6 nM fluorescence sequence in the same saline buffer. Sequences are provided in Fig. 2. Both solutions were incubated for 30 min at 37°, protected from light.

### Fabrication of CellStudio substrates

To generate CellStudio substrates consisting of combined patterns of microbeads and cells, printing and vacuum lithography (PnVlitho) was performed, as previously described [18] Polydimethylsiloxane (PDMS) slabs (Ellsworth Adhesives, Spain) containing channel-like structures (1000 × 5000 × 13 µm, width × length × height) with internal pillars (100 µm diameter, separated by 100 µm) were used. The PDMS slabs were punched twice to create a 2-mm inlet and a 1-mm outlet for fluid flow.

A 10 µL droplet of a 50 µg mL⁻^1^ fibronectin solution (Fisher Scientific, Spain) in 1X phosphate-buffered saline (PBS) (Sigma-Aldrich, Spain) was applied to the pillar-containing area of the PDMS channels and incubated for 30 min. After incubation, the PDMS slabs were rinsed with distilled water, dried with compressed air, and brought into contact with 35 mm glass-bottom dishes (MatTek, USA). Prior to this, the glass substrates were oxidized in an air plasma chamber (29.6 W for 60 s, BlackHole, France). The resulting PDMS-glass assembly was placed under vacuum (0.7 mbar) inside a desiccator for 20 min.

Next, the outlets were sealed with tape, and 2 µL of a 6 × 10^11^ microbeads mL⁻^1^ suspension of 200 nm diameter streptavidin-coated polystyrene microbeads (Bangs Laboratories, USA) was introduced into the inlets. Sealing the outlet of each channel was necessary to prevent phase separation of the bead suspension during flow and to ensure uniform distribution of microbeads inside the channels. The suspension was allowed to flow until it reached the outlet. After 5 min, the tape was removed, and the PDMS-glass assembly was stored overnight at 4 °C to allow solvent evaporation. Finally, the PDMS slabs were removed, revealing microbead patterns surrounding the fibronectin adhesion dots.

To prevent non-specific adsorption, the substrates were treated with 1 mL of a blocking solution containing 5% Bovine Serum Albumin (BSA) (Sigma-Aldrich, Spain), 10% casein (Fisher Scientific, Spain), and 0.2% milk powder in PBS. The substrates were incubated with the blocking solution for 2 h. After incubation, the blocking solution was removed, and the wells were rinsed three times with PBS to ensure thorough washing.

For microbead pattern functionalization, VEGF-SSSA solutions were added to the CellStudio substrates and incubated for 1 h at room temperature, protected from light, to facilitate binding of the aptamer construct to the microbeads via biotin-streptavidin interactions. After incubation, the solution was removed, and the wells were rinsed three times with PBS to ensure proper washing.

### Validation of VEGF-SSSA assay: quenching performance and sensitivity on CellStudio

To evaluate the performance of the VEGF-SSSA assay on the CellStudio substrates, two experiments were conducted. First, the quenching efficiency of the VEGF-SSSA was assessed by comparing the fluorescence intensities of substrates incubated with three conditions: the VEGF-SSSA-Max_fluo_ solution (lacking the quencher sequence), the fully assembled VEGF-SSSA solution, and the VEGF-SSSA solution incubated with 1000 ng mL⁻^1^ vascular endothelial growth factor 165 (VEGF, Fisher Scientific, Spain). Following incubation and three PBS rinses, the substrates were directly imaged using fluorescence microscopy.

To assess the sensitivity of the VEGF-SSSA assay, the CellStudio substrates were first incubated with the VEGF-SSSA solution as described in the “VEGF-SSSA solutions” section. The substrates were then incubated for 1 h with VEGF solutions at increasing concentrations (0, 1, 10, 100, and 1000 ng mL⁻^1^). Following incubation, the solutions were removed, and the substrates were rinsed three times with PBS. After rinsing, fluorescence microscopy was used to directly image the substrates.

### Cell seeding and VEGF secretion analysis on CellStudio substrates

For mesenchymal stem cells patterning, hair human follicle mesenchymal stem cells (hHF-MSCs, isolated from human donors’ follicles) were detached from the flasks and were resuspended in serum free Dulbecco’s Modified Eagle’s medium (SFM, Fisher Scientific, Spain) at a concentration of 10^5^ cells mL^−1^. A total of 750 µL of the cell suspension was loaded into the substrates modified with the VEGF-SSSA. To ensure specific cell adhesion to the fibronectin dots, the wells were left inside the incubator (37 °C, 5% CO_2_) on constant oscillation using a rocker (Vari-Mix steep angle rocker, Thermo Fisher) for a maximum time of 120 min. Afterwards, the medium was retrieved, the wells were rinsed three times with PBS to wash out any non-attached cell. For cell maintenance during the course of the experiments, cell-loaded substrates were incubated with 750 µL of SFM. Cells were then maintained secreting for a period of 24 h inside the incubator avoiding movement. Afterwards, the substrates were immediately imaged through fluorescence microscopy.

### Image and data analysis

Brightfield and fluorescence microscope images were taken with an Olympus inverted CKX53 microscope (Japan), coupled with a DP23M monochrome camera and LED illumination CKX3-RFA with EM filters DAPI-FITC-TRITC-Cy5.

For the analysis of the fluorescence intensity in the immediacy of each spot, a ring-circular ROI was used (diameter of 100 and 110 µm, inner circle and outer circle, respectively) that covered from the edge of the spot up to 10 µm away from it. A single data point was obtained for each cluster by calculating the mean fluorescence intensity within this ROI. Microscopy images were processed by FiJi/ImageJ software. Data and statistical analysis were performed in Excel 2016 and GraphPad Prism 8.

## Results and discussion

### Performance of the VEGF-SSSA

CellStudio substrates were developed using a combination of microcontact printing and vacuum lithography (PnVlitho), a technique previously established by our group [23]. CellStudio substrates consist of a two-dimensional pattern of protein dots surrounded by a three-dimensional microbead network. This configuration enables spatial control over cell attachment, promoting the formation of uniformly spaced cell clusters in direct interaction with the surrounding microbeads. Each CellStudio substrate accommodates hundreds of individual small cell clusters, serving as independent replicates. In this study, PDMS stamps with uniformly spaced pillars were used to generate arrays containing 250 protein dots per substrate.

The microbeads function as carriers of structure-switching signaling aptamers (SSSA), enabling single-step detection of live-cell secreted factors. SSSA are double-stranded DNA sequences that incorporate an aptamer capable of recognizing a specific target molecule. Upon target binding, a conformational change triggers strand displacement of a quencher sequence, leading to fluorescence activation. Previously, our group developed a VEGF-specific SSSA (VEGF-SSSA) based on the 3R02 aptamer, which was successfully used to detect VEGF secretion from mesenchymal stem cells (MSCs) using fluorescence analysis on a paper microfluidic device (µPAD) [29]. By functionalizing the microbeads within the CellStudio platform with the VEGF-SSSA, it becomes possible to detect VEGF secretion directly from live-cells in the proximity of hundreds of individual cell clusters within a single substrate.

The VEGF-SSSA, depicted in Fig. 2A, consists of three key components. The first component is the aptamer sequence, which includes a biotin molecule at its 3′ end to facilitate binding to streptavidin-coated microbeads. This sequence contains the 3R02 aptamer, responsible for VEGF recognition, located between nucleotides 22 and 46 (3′−5′). The second component is a fluorescence sequence, complementary to the aptamer at nucleotides 1 to 15, and labeled with a Cy5 fluorophore at its 3′ end. The third component is a quencher sequence, which is complementary to the aptamer at nucleotides 17 to 29 and partially overlaps with the 3R02 recognition site. This sequence includes an Iowa Black quencher at its 5′ end. When all three sequences are hybridized, the one-nucleotide gap between the fluorescence and quencher sequences ensures efficient quenching of the Cy5 signal. Upon VEGF binding, the quencher sequence is displaced, allowing fluorescence emission.

To evaluate the quenching efficiency and VEGF detection capability of the VEGF-SSSA after immobilization on microbead patterns, we analyzed fluorescence intensity under three different conditions. First, we examined the unquenched VEGF-SSSA, in which only the aptamer and fluorescence sequences were present, and the quencher sequence was absent. In this state, the fluorophore remained fully exposed, allowing maximum fluorescence emission (Max_fluo_). Next, we tested the quenched VEGF-SSSA, a fully assembled structure with all three sequences, where the quencher sequence was hybridized to the aptamer, effectively suppressing fluorescence emission (Q_fluo_). Finally, we evaluated the fluorescence emission of the quenched VEGF-SSSA following incubation with 1000 ng mL⁻^1^ VEGF.

After confirming the successful immobilization of VEGF-SSSA in both states, we compared fluorescence intensities across these conditions (Fig. 2B). The difference in fluorescence intensity between Max_fluo_ and Q_fluo_ was 730%, demonstrating the system’s large dynamic range. Finally, when quenched VEGF-SSSA substrates were incubated with VEGF, fluorescence intensity increased by 85 ± 15% compared to Q_fluo_, indicating successful displacement of the quencher sequence upon VEGF recognition. These results confirm the system’s ability to differentiate between distinct states of the VEGF-SSSA and its applicability for detecting VEGF secretion within the CellStudio platform.

### Sensitivity of the VEGF-SSSA assay in microbead patterns

To evaluate the sensitivity and dynamic range of the VEGF-SSSA assay for detecting VEGF secretion from patterned small cell clusters, we tested its performance on CellStudio substrate over different concentrations of VEGF. For this, CellStudio substrates composed of streptavidin-coated microbeads (200 nm in diameter) patterned around empty fibronectin circular regions (100 µm in diameter) were functionalized with biotinylated VEGF-SSSA. The substrates were then incubated with increasing concentrations of VEGF (0, 1, 10, 100, and 1000 ng mL⁻^1^) for 1 h, followed by direct fluorescence imaging using fluorescence microscopy. Fluorescence intensity was quantified in the immediate surroundings of each dot by applying a 10-µm circular region of interest (ROI).

**Fig. 3:**
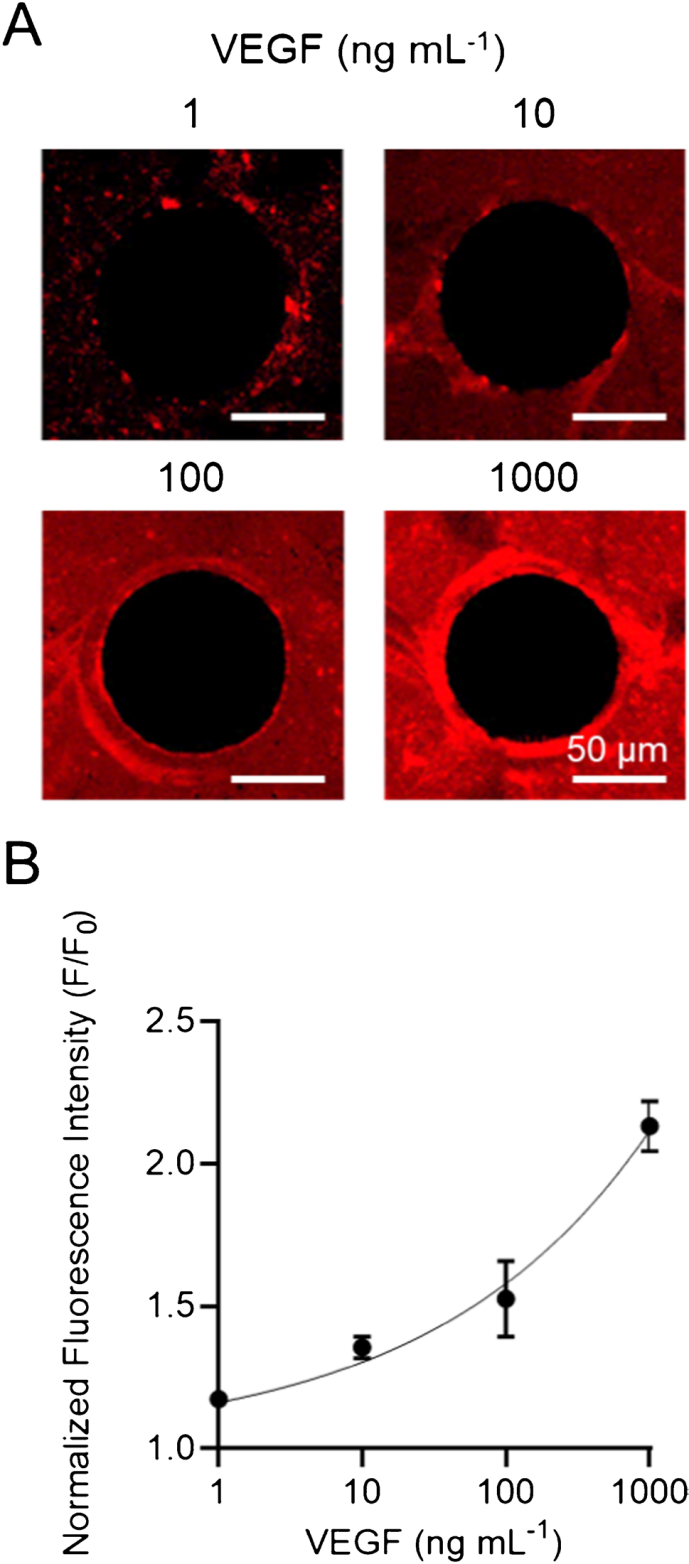
Analysis of VEGF-SSSA sensitivity. **A** Fluorescence images of microbead patterns surrounding a non-functionalized circular area (black dot), functionalized with VEGF-SSSA (red), after incubation with increasing concentrations of VEGF (1, 10, 100, and 1000 ng mL⁻^1^). **B** Plot of the normalized fluorescence intensity measured within the 10 μm ring-shaped region of interest (ROI). Fluorescence values were normalized to the mean intensity of the quenched VEGF-SSSA (0 ng mL⁻^1^). Error bars represent the standard error of the mean (SEM); data collected from 100 dots across 4 samples

A clear trend of increasing fluorescence intensity was observed with increasing VEGF concentrations, confirming the sensitivity of the VEGF-SSSA assay within the microbead patterns (Fig. 3). Relative to the quenched state of the VEGF-SSSA (0 ng mL⁻^1^), fluorescence intensity in the areas surrounding each dot increased by 18% ± 11, 35% ± 3, 52% ± 13, and 113% ± 10 for VEGF concentrations of 1, 10, 100, and 1000 ng mL⁻^1^, respectively. These results demonstrate the assay’s ability to detect VEGF across a wide range of concentrations, reinforcing its sensitivity for different VEGF levels. Furthermore, the dynamic range of the assay suggests it can potentially accommodate the broad spectrum of VEGF secretion levels observed across different cellular contexts, which vary based on external stimuli, microenvironmental conditions, and specific phases of the cell cycle [30–32].

The data were fitted to a four-parameter logistic (4PL) regression curve, resulting in a calculated limit of detection (LOD) of 1.4 ng mL⁻^1^. This detection limit represents a 100-fold improvement over the previously reported microPAD platform [29], which exhibited an LOD of 137 ng mL⁻^1^, which relied on extended bulk supernatant collection from cell cultures and lacked spatial resolution. Our system enables detection of VEGF quantities as low as 0.002 picograms within the area corresponding to a single protein dot, approximately 5000 times lower than the total mass required in a standard ELISA well [33]. The enhanced sensitivity of the CellStudio platform is attributed to the precise spatial localization of biosensors within the microbead patterns and the improved fluorescence contrast between the bright bead regions and the dark fibronectin-free areas. These factors significantly increase the signal-to-noise ratio, enabling the detection of lower VEGF concentrations with greater accuracy.

### Live-cell detection of secreted VEGF from patterned MSCs-clusters

Building on the demonstrated sensitivity of the VEGF-SSSA for VEGF detection, we applied this aptasensor to monitor VEGF secretion from small MSC clusters. In this context, a cell cluster is defined as a confined group of 4–5 mesenchymal stem cells adhered to an individual fibronectin-patterned dot. To achieve this, CellStudio substrates composed of streptavidin-coated microbeads (200 nm in diameter) patterned around cell-adhesion fibronectin dots (100 µm in diameter) were functionalized with VEGF-SSSA. MSCs were then seeded onto the platform and incubated for 2 h, allowing them to form the small clusters. Cells were cultured for 24 h to facilitate VEGF secretion, after which fluorescence imaging was performed directly on the live patterns using fluorescence microscopy.

**Fig. 4:**
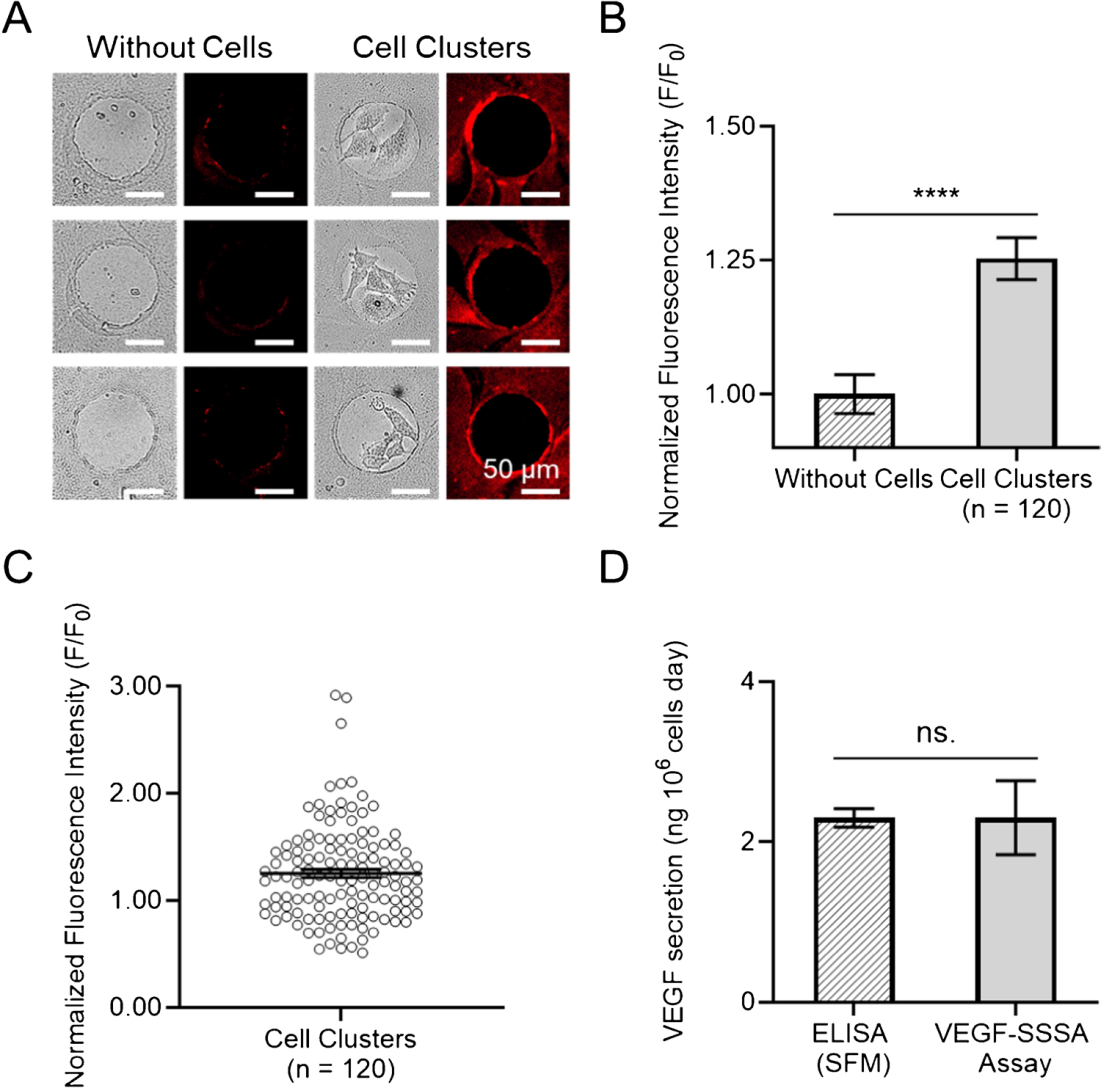
Live-cell, high-throughput detection of VEGF secreted by cell clusters on combined cells and microbeads patterned substrate. **A** Brightfield and fluorescence images of six different areas of a substrate, comprised of three fibronectin dots without cells (left) and three with cells (right) surrounded by patterned microbeads functionalized with the VEGF-SSSA (red), after 24 h incubation. **B**, **C** Bar plot and dispersion plot of the normalized fluorescence intensity of a ring-shaped ROI of 10 µm around the dots without cells and the cell clusters. All data was normalized with the mean value of the corresponding quenched VEGF-SSSA (Without Cells). Error bars mean SEM (*n* = 100 dots from three samples). Statistical significance: non-parametric Mann–Whitney test (****p ≤ 0.001). **D** Plot of VEGF secretion analyzed through ELISA and the VEGF-SSSA assay on the CellStudio substrate. Statistical significance: non-parametric Mann–Whitney test (ns. *p* > 0.05)

Fluorescence intensity in the microbeads surrounding each MSC cluster was significantly higher than in negative control conditions without cells, confirming VEGF secretion and its recognition by the immobilized VEGF-SSSA (Fig. 4A). Quantitative image analysis revealed a 25% increase in mean fluorescence intensity compared to negative control regions consisting of fibronectin-patterned dots without attached cells (Fig. 4B). A low level of background fluorescence was observed in cell-free regions, which can be attributed to incomplete quenching of the fluorophore; however, this residual signal remains significantly lower than the fluorescence detected around VEGF-secreting clusters. While VEGF secretion rates exhibited high heterogeneity among individual cell clusters (Fig. 4C), the large number of analytical points enabled by the platform ensures strong statistical significance in the measurements. This variability could arise from a combination of factors, including intrinsic cell-to-cell heterogeneity, differences in the number of cells per fibronectin dot, and small variance in beads distribution. This measurement corresponded to an estimated localized VEGF concentration of 3 ± 1 ng mL⁻^1^ and a secretion rate of 1.5–3 ng per 10⁶ cells per day. These findings are consistent with VEGF secretion quantifications obtained from batch MSC cultures using ELISA (Fig. 4D) and align with our previous studies utilizing a commercial VEGF immunoassay on CellStudio substrates [22]. The close proximity of the microbeads to the cell clusters promotes localized accumulation of secreted proteins, while the three-dimensional structure of the beads further contributes by offering an increased surface area for protein capture. The ability to perform live-cell imaging while preserving the cellular microenvironment offers a significant advantage, potentially allowing for simultaneous monitoring of both cellular activity and secretion events.

**Fig. 5:**
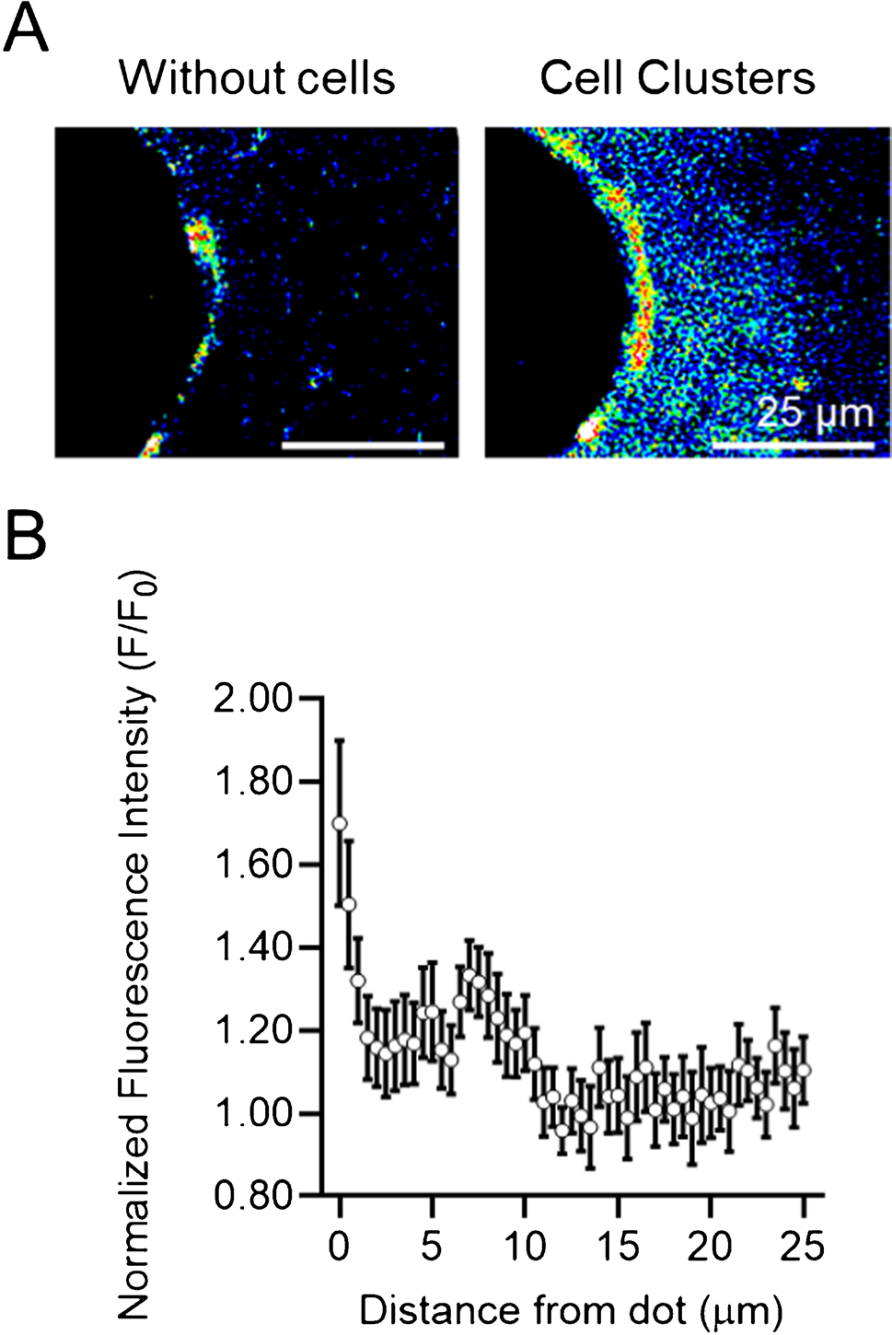
Diffusion of VEGF secretion from cell clusters. **A** Fluorescence images (heat gradient) of a region of a CellStudio substrate showing a protein dot without cells (top) and with cells (bottom), illustrating VEGF diffusion from the dot outward. **B** Plot of the normalized fluorescence intensity measured in cell clusters at 0.5-µm intervals from the edge of the dot up to 25 µm. Fluorescence values were normalized to the mean intensity of the corresponding quenched VEGF-SSSA (without cells). Error bars represent the standard error of the mean (SEM); data collected from 10 dots across 3 samples

Furthermore, the CellStudio platform enables spatially resolved analysis of secretion, allowing for the evaluation of the adsorption and diffusion of secreted VEGF from each cell cluster across the culturing substrate. To assess this, the fluorescence intensity gradient was measured from the edge of the fibronectin dots outward to a distance of 25 µm (Fig. 5). Fluorescence analysis showed that intensity was significantly higher within 10 µm of each cell cluster compared to controls without cells, with a gradual decrease as the distance from the dot increased. Notably, at distances between 15 and 25 µm, fluorescence levels were not significantly different from those observed in negative controls. These findings confirm that the most secreted VEGF remains localized near each cell cluster and that the observed fluorescence intensity around the dots primarily originates from VEGF secretion by the corresponding cluster. This highlights the potential of combining CellStudio substrates with SSSA assays to study secretion dynamics at high spatial resolution.

By precisely defining the analysis area, this approach allows for a more accurate assessment of secretion activity. Furthermore, by adjusting both SSSA and bead concentrations, adsorption and retention can be modulated, enabling the study of spatially resolved diffusion patterns under different cellular conditions. This capability opens the door to rapid and straightforward analyses of cell–cell communication mediated by soluble factor secretion.

## Conclusions

This study demonstrates the successful integration of a self-reporting VEGF-SSSA biosensor into the CellStudio platform, enabling single-step detection of VEGF secretion from live MSC clusters without the need of additional reagents or sample collection. The assay exhibited a limit of detection (LOD) of 1.4 ng mL⁻^1^, representing a 100-fold increase in sensitivity compared to previous reports from our group, which relied on extended bulk supernatant collection from cell cultures and lacked spatial resolution. By localizing the aptasensors in close proximity to the patterned cell clusters, the system enables rapid, spatially resolved detection of protein secretion within just 24 h. Furthermore, its performance was validated against conventional techniques, confirming its reliability for sensitive VEGF detection across a wide dynamic range.

This work advances our previous CellStudio reports by enabling non-invasive, live-cell secretion monitoring while allowing simultaneous direct cell observation. Detecting secretion from hundreds of clusters made of just 4–5 cells ensures high experimental throughput, while eliminating fixation steps enables more physiologically relevant studies of cell behavior and secretion dynamics over time.

Beyond its current capabilities, the integration of SSSA biosensors into CellStudio presents the potential for real-time secretion monitoring. With further optimization of SSSA kinetics and fluorescence signal dynamics, this approach could evolve into a platform capable of continuous monitoring of secretion events using standard fluorescence microscopy, offering high temporal resolution while maintaining spatial specificity.

The modular and user-friendly design of the CellStudio platform facilitates seamless integration into existing laboratory workflows, making it a versatile tool for researchers in biomedical sciences, tissue engineering, and drug discovery. Future developments, such as the functionalization of microbeads with additional aptasensors and environmental modulators, could further expand its capabilities. In addition, the platform’s adaptability allows for the incorporation of diverse surface chemistries and extracellular matrix (ECM) components, enabling precise studies of cell–ECM and cell–cell interactions. By leveraging microbeads as carriers not only for biosensors but also for signaling molecules or matrix fragments, CellStudio opens new possibilities for detecting a broader range of biomolecules and for modulating the local cellular environment.

## Data Availability

The authors declare that the data supporting the findings of this study are available within the paper. Should any raw data files be needed in another format they are available from the corresponding author upon reasonable request.
