# Correction: Non-invasive detection of VEGF secretion from small clusters of mesenchymal stem cells using VEGF-SSSA integrated into the CellStudio platform

**DOI:** 10.1007/s00604-025-07581-4

**Published:** 2025-10-01

**Authors:** Enrique Azuaje-Hualde, Naiara Lartitegui-Meneses, Fernando Benito-Lopez, Lourdes Basabe-Desmonts

**Affiliations:** 1 Microfluidics Cluster UPV/EHU, BIOMICs Microfluidics Group, University of the Basque Country UPV/EHU, Vitoria-Gasteiz, Spain; 2 Microfluidics Cluster UPV/EHU, Analytical Microsystems & Materials for Lab-On-a-Chip (AMMa-LOAC) Group, University of the Basque Country UPV/EHU, Leioa, Spain; 3 Basque Foundation of Science, IKERBASQUE, Bilbao, Spain

**Correction: Microchimica Acta (2025) 192:484**

10.1007/s00604-025-07319-2

In this article the author’s name 'Naiara Lartitegui-Meneses' was incorrectly written as 'Naiara Lartitegi'.

The original article has been corrected.

